# Nephrolithiasis

**Published:** 1905-10-07

**Authors:** 


### NEPHROLITHIASIS.

Stone in the kidney is an ailment which has brought a good deal of discredit upon the members of the medical profession in recent years. In this particular department medical men have no cause for self-satisfaction. The mistakes are almost incessant. Stone is diagnosed when no stone exists, and operations are performed which bring no relief, but occasionally have dire results. It is probably near the truth to say that up to the present time renal surgery has been rather a curse than a blessing to mankind. In spite of this nine out of ten surgeons still retain their faith in the therapeutic powers of the knife in renal cases.

Dr. Howard Lilienthal,[1] in a paper read before the Harvard Medical Society of New York City, refers to the failures of operations on the kidney and draws attention to the success which frequently attends less drastic measures. He is of opinion that when only one or two attacks of renal colic have occurred it is well to give the patient a thorough trial of what he calls the water treatment. In this the patient drinks a quart of water four times a day, each quart being taken at a sitting of not longer than fifteen minutes. As an example of the success of this treatment he mentions the case of a young man of thirty-two years, who had the usual characteristic symptoms of renal colic followed by profuse hæmaturia. He suffered for an hour before being seen and passed almost pure blood. After a hot bath and rest for some hours, the attack subsided, and the patient could get about next day. He was directed to take four quarts of water between meals. Over a year later a stone was passed, and the patient has been well ever since.

During an attack of renal colic the patient should be advised to lie down with the foot of the bed or couch raised, so that the hips are higher than the body; in this way an attack may be cut short—a statement which was endorsed by Dr. Cabot in the course of the discussion which followed the reading of Dr. Lilienthal's paper. Among the sources of error in diagnosis are the fallibility of $x$-ray photographs, hysterical renal colic, movable kidney, crystaluria, etc. With regard to operation, this should be reserved for cases where there have been several attacks of renal colic and the patient is unrelieved by the water treatment, where the stone is very large, and where pyonephrosis is present. If many stones are found to be present when the kidney is exposed, Dr. Lilienthal thinks it is better to remove the kidney than to do nephrotomy. With regard to anuria following operation or an attack of renal colic, he points out that the cause may be organic or reflex. The only active kidney may have become obstructed by a stone, or rendered inactive by manipulation or other surgical injury, or else the anuria may be of a reflex nature; interference with one kidney causing temporary suspension of activity in the other. In either case time will clear up the diagnosis. Patients will live from seven to ten days without passing a drop of urine. So it is better to assume that anuria following renal colic or operation upon one kidney is of a reflex and temporary nature, than to act upon the assumption that it must be due to obstruction or to the permanent cessation of function of a solitary kidney. In one of Dr. Lilienthal's cases, an examination by the uretoscope was followed by complete anuria lasting for forty-eight hours, after which there was a free flow of urine. In this instance there can be little doubt that the anuria was of a reflex nature, and to hove treated it by immediate operation as a case of obstructive anuria would have been disastrous.

[1] Medical News, September 16, 1905.

